# The “Alloun” Maneuver: A New Technique for Repositioning Difficult-To-Access Renal Stones During Endoscopic Treatment

**DOI:** 10.7759/cureus.96272

**Published:** 2025-11-07

**Authors:** Jihad El Anzaoui, Ali Akjay, Najwa Jmil, Abdessamad El Bahri, Mohammed Lezrek

**Affiliations:** 1 Urology, Urology, Mini-Invasive Surgery, Robotic Surgery, Artificial Intelligence, and Pedagogical Innovations Laboratory, Faculty of Medicine, Sidi Mohamed Ben Abdellah University, Fez, MAR; 2 Urology, Moulay Ismail Military Hospital, Meknes, MAR; 3 Urology, Mohammed V Military Hospital, Rabat, MAR; 4 Urology, Moulay Ismail Hospital, Meknes, MAR

**Keywords:** flexible ureterorenoscopy, laser holmium, percutaneous nephrolithotomy (pcnl), renal stone surgery, thulium fiber laser

## Abstract

Flexible renoureteroscopy and percutaneous nephroscopy are both the gold standards to treat kidney stones. The success rate of these techniques depends mainly on the location of the stone.

In some challenging positions, access to the stone is impossible, indicating the failure of the whole procedure. Striving to access a stone in a challenging position can expose the endoscope to a high level of torsion that can damage it.

We propose a new technique of stone repositioning applicable during percutaneous nephrolithotomy or flexible ureteroscopy using a laser fiber of any diameter. This technique is particularly useful when the stone is only partially accessible. We have developed this simple, easy, and cost-effective trick to minimize our rate of procedure failure and to prevent material forced torsion.

## Introduction

The anatomy of the pelvicalyceal system plays a crucial role in determining the stone-free rate following endoscopic management of renal calculi [1,2]. Achieving complete clearance often depends on optimal access to all calyceal regions. However, gaining access through the renal pelvis or a calyceal fornix does not always allow comprehensive visualization and instrumentation of the entire collecting system. Therefore, the pelvicalyceal anatomy can be particularly challenging, especially in cases with long, narrow, or acutely angulated infundibula, which significantly limit endoscopic maneuverability and fragment retrieval [3]. These anatomic constraints are frequently encountered in daily endourological practice and may reduce treatment efficacy. As a result, surgeons may need to perform multiple procedures or create additional percutaneous tracts during percutaneous nephrolithotomy (PCNL) to achieve complete stone clearance, which can be time-consuming, occasionally risky, and may require additional devices.

## Technical report

Methods

We describe a new technique that can be applied in either flexible ureterorenoscopy (FURS) or PCNL using a laser fiber. This technique involves repositioning a renal stone that the scope can only partially access.

Fragmenting the stone in this position risks deeper migration. Our method allows for the mobilization of the stone by creating a hole in it. Perforating the stone enables the laser fiber tip to be embedded in it, and then a lateral displacement of the laser fiber moves the stone from the targeted area to the desired area (Video 1, Video 2).

**Video 1:** Alloun technique 1 “Alloun” technique practiced on a renal stone of a lower calyx.

**Video 2:** Alloun technique 2 “Alloun” technique permitting an easy and ample mobilization of a pelvic renal stone.

At the beginning of the experience, this technique was used in 20 cases of flexible ureteroscopy and 20 cases of PCNL as shown in the video. Currently, this is used routinely in our practice.

Results

Three key factors contribute to the success of the “Alloun” technique.

Diameter of the Fiber

A thinner fiber diameter allows for a more precise digging, minimizing edge fragmentation. Perforation continues until the fiber can move the stone laterally. With very thin fibers, the section embedded in the stone may weaken and break during mobilization. In such cases, the fragment can be retrieved with forceps or a basket. We preferably use a fiber, the core diameter of which is between 300µm and 600µm. Breaking of the fiber tip is never a barrier to continuing the mobilization of the stone. The likelihood of fiber breakage depends on factors such as the fiber diameter, the hardness and burden of the stone, and the dexterity of the operator.

Laser Settings

For both holmium and thulium lasers, the fragmentation mode (high energy, low frequency, approximately 10 W power, short-pulse mode) is preferable to the dusting mode, as it allows the creation of an easy and clean hole inside the stone.

Width of the Desired Area

This parameter appears to be the most critical determinant of maneuver success. When the desired area, including the entrance to the calyx, is wider than the diameter of the stone and can accommodate it, mobilization of the stone is always possible, with a success rate of 100%. Ideally, the space should be sufficiently wide to stabilize a substantial portion of the stone during fragmentation. In smaller spaces, there is a higher risk of mucosal trauma and bleeding during stone mobilization (Video 3). Thus, careful selection based on anatomical compatibility is essential for optimal outcomes. In our series, all mucosal traumas were superficial and bleeding was manageable. After mobilization and stabilization of the stone, lithotripsy may cause subsequent migration of the stone or fragments. Ideally, fragmentation should be modulated to minimize retropulsion and prevent migration.

**Video 3:** Alloun technique 3 The mobilization of the stone in short desired area can cause bleeding of the mucosa.

## Discussion

During FURS, the scope may struggle to reach some angulated areas of the pelvicalyceal system. Extreme deflection of the scope can prevent the laser fiber from coming out of the operator's canal. Introducing the laser fiber reduces the scope’s flexibility, sometimes severely limiting access to difficult locations.

Several techniques have been developed to simplify renal stone access and lithotripsy [4]. Repositioning lower calyx stones to a more accessible position is a well-known method. Injecting saline into the targeted location can also help flush out entrapped stones.

Moreover, applying proximal torsion to the scope using the index and thumb of the non-dominant hand can improve side deflection but increases the risk of scope dysfunction.

In PCNL, using multiple tracts is a valid and safe option to enhance the stone-free rate in complex cases; however, it may increase the risk of bleeding and renal function impairment [5].

In PCNL, a flexible nephroscope can reach more challenging areas, but it is costly. Combined surgery using both a rigid nephroscope percutaneously and a retrograde flexible ureteroscope is an effective but time-consuming and expensive procedure. Interestingly, some surgeons have developed simpler maneuvers to displace stones from inaccessible areas [6]. Puncturing the targeted calyx without dilation can flush the stone with a simple saline injection, while precutting the flank can sometimes move stones from anterior calyces to more favorable locations.

**Figure 1:**
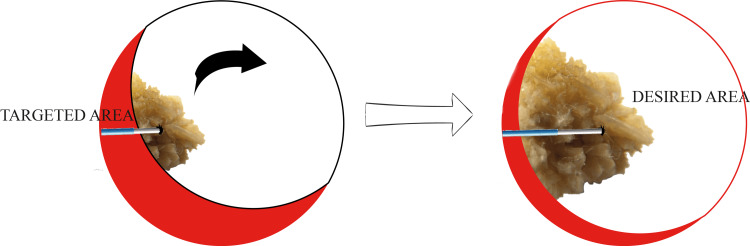
Representation of an endoscopic view by nephroscopy or ureterorenoscopy Representation of an endoscopic view showing a laser fiber perforating a partially accessible renal stone, which allows the mobilization of the stone from the targeted area to the desired and more favorable area. This is an original image.

Our technique, which involves mobilizing the stone from the targeted area to a more favorable location (desired area), helps avoid excessive torsion of the scope and allows for comfortable fragmentation with easier fragment retrieval (Figure 1).

**Figure 2:**
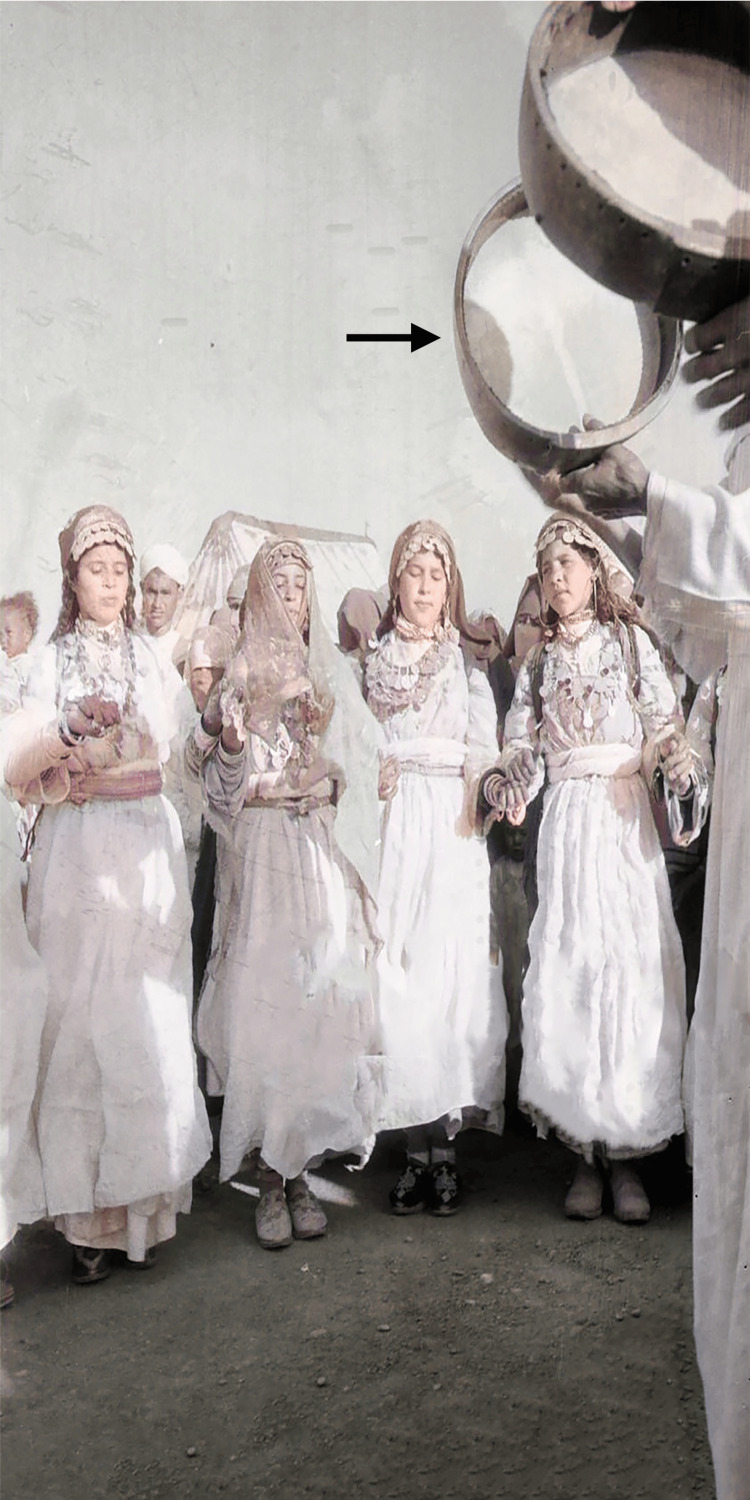
Dancers performing with the Alloun Amazigh dancers from Morocco’s Atlas Mountains performing with the Alloun (arrow) during a concert in 1960. Licensed courtesy of Getty Images [8].

**Figure 3:**
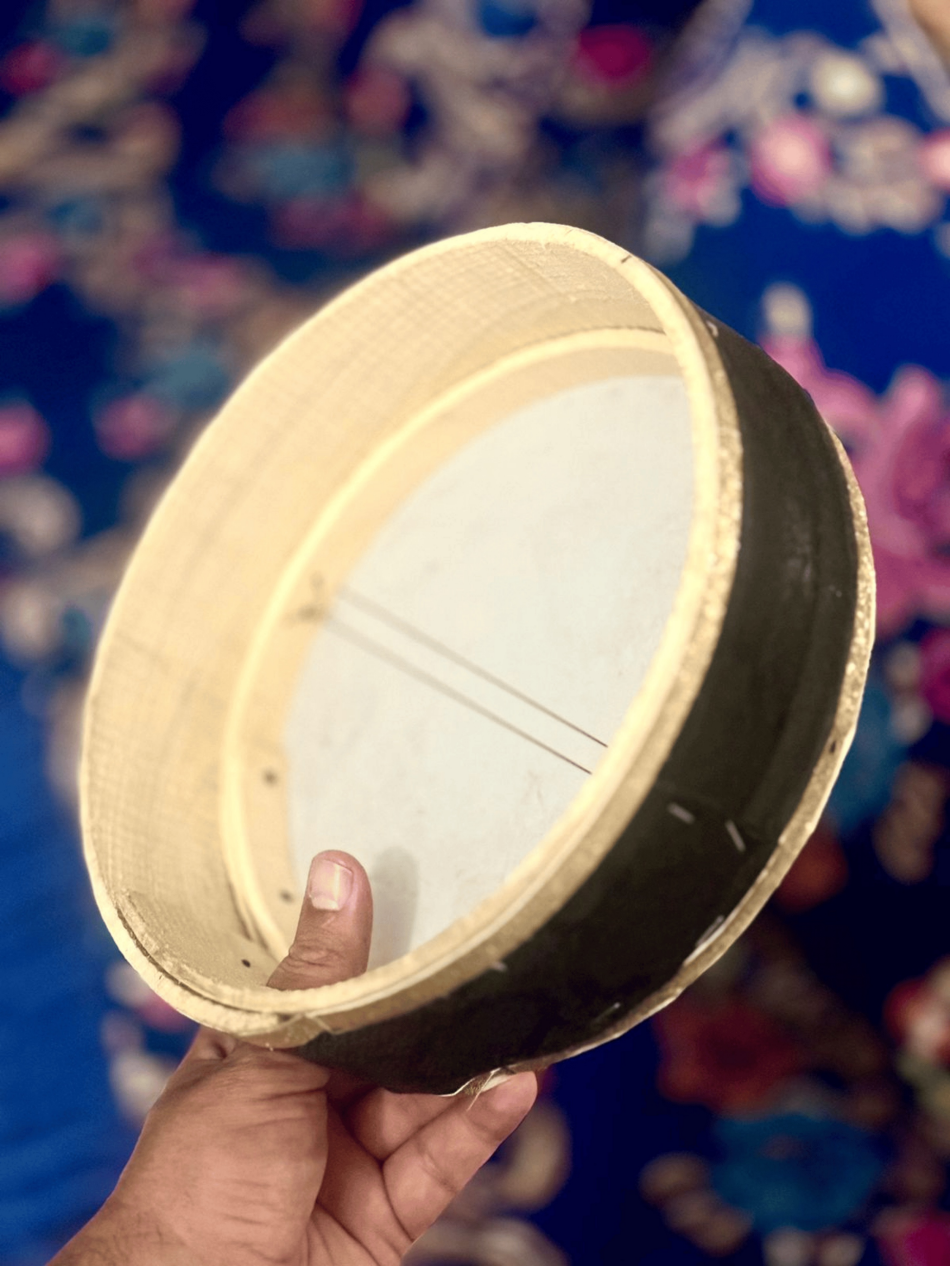
The Alloun instrument The left thumb uses the lateral hole to manipulate the instrument. This is an original image

"Alloun" is an Amazigh noun of an ancient musical instrument widely used in the Maghreb, consisting of a frame and drum [7]. The drum is played vertically by inserting the left thumb into a special hole in the frame. This thumb insertion in the hole is crucial for balancing and manipulating the instrument, allowing for easy mobilization while the right hand applies percussion on the drum (Figures 2, 3). The "Alloun" technique illustrates how a hole in the stone can be used to move it without excessive effort.

## Conclusions

In PCNL or FURS, certain stone locations can present significant challenges. When a stone is only partially accessible, the “Alloun” technique provides a safe and effective method for repositioning it into a more favorable area, thereby facilitating easier fragmentation and extraction.

This practical tip can enhance surgical efficiency and improve stone-free rates while minimizing the need to resort to more complex or risky maneuvers. Prospective studies with more elaborated quantitative outcome data are needed to fully establish the value and generalizability of this technique.
